# Cepabiflas B and C as Novel Anti-Inflammatory and Anti-Apoptotic Agents against Endotoxin-Induced Acute Kidney and Hepatic Injury in Mice: Impact on Bax/Bcl2 and Nrf2/NF-κB Signalling Pathways

**DOI:** 10.3390/biology12070938

**Published:** 2023-06-30

**Authors:** Akaber T. Rizq, Alaa Sirwi, Dina S. El-Agamy, Hossam M. Abdallah, Sabrin R. M. Ibrahim, Gamal A. Mohamed

**Affiliations:** 1 Department of Natural Products and Alternative Medicine, Faculty of Pharmacy, King Abdulaziz University, Jeddah 21589, Saudi Arabia; aasaadrizq@stu.kau.edu.sa (A.T.R.); asirwi@kau.edu.sa (A.S.); hmafifi@kau.edu.sa (H.M.A.); 2 Department of Pharmacology and Toxicology, Faculty of Pharmacy, Mansoura University, Mansoura 35516, Egypt; dinaagamy@mans.edu.eg; 3 Department of Chemistry, Preparatory Year Program, Batterjee Medical College, Jeddah 21442, Saudi Arabia; sabrin.ibrahim@bmc.edu.sa; 4 Department of Pharmacognosy, Faculty of Pharmacy, Assiut University, Assiut 71526, Egypt

**Keywords:** cepabiflas B and C, *Allium cepa*, endotoxemia, hepatorenal injury, LPS, Nrf2/NF-κB, Bax/Bcl2, health and wellbeing, sustainable development goals, waste management

## Abstract

**Simple Summary:**

Endotoxin liver/kidney injury is characterized by oxidative stress, inflammation, and apoptosis that results in acute hepatic and kidney dysfunction with limited effective intervention. This study evaluated the potential protective efficacy of cepabiflas B and C (CBs) separated from Allium cepa against LPS (lipopolysaccharide)-induced hepatic and kidney damage and its possible mechanistic pathways. CBs effectively protected the liver and the kidney against LPS-induced damage. CBs ameliorated oxidative damage and enhanced Nrf2/HO-1 protective pathway. In reverse, CBs counteracted the activation of NF-κB/downstream cytokines which attenuated LPS-induced inflammatory response. CBs showed remarkable anti-apoptotic activity as it enhanced Bcl2 and suppressed Bax/caspase-3. Hence, this study elucidated the new therapeutic use of CBs in septic patients.

**Abstract:**

Cepabiflas B and C (CBs) are flavonoid dimers separated from *Allium cepa*. They demonstrated antioxidant and α-glucosidase and protein tyrosine phosphatase 1B inhibition capacities. However, their anti-inflammatory activities and their effects on endotoxemia are unknown. The current study aimed at exploring the protective activities of CBs on lipopolysaccharide (LPS)-induced kidney and liver damage in mice and investigating the possible molecular mechanisms. Mice were orally treated with a low (40 mg/kg) or high (60 mg/kg) dose of CBs for five days prior to a single intraperitoneal injection of LPS (10 mg/kg). Samples of serum and hepatic and kidney tissues were collected 24 h after the LPS challenge. Changes in serum indices of hepatic and renal injury, pathological changes, molecular biological parameters, and proteins/genes related to inflammation and apoptosis of these organs were estimated. LPS injection resulted in deleterious injury to both organs as indicated by elevation of serum ALT, AST, creatinine, and BUN. The deteriorated histopathology of hepatic and renal tissues confirmed the biochemical indices. CBs treated groups showed a reduction in these parameters and improved histopathological injurious effects of LPS. LPS-induced hepatorenal injury was linked to elevated oxidative stress as indicated by high levels of MDA, 4-HNE, as well as repressed antioxidants (TAC, SOD, and GSH) in hepatic and kidney tissues. This was accompanied with suppressed Nrf2/HO-1 activity. Additionally, there was a remarkable inflammatory response in both organs as NF-κB signalling was activated and high levels of downstream cytokines were produced following the LPS challenge. Apoptotic changes were observed as the level and gene expression of Bax and caspase-3 were elevated along with declined level and gene expression of Bcl2. Interestingly, CBs reversed all these molecular and genetic changes and restricted oxidative inflammatory and apoptotic parameters after LPS-injection. Collectedly, our findings suggested the marked anti-inflammatory and anti-apoptotic activity of CBs which encouraged its use as a new candidate for septic patients.

## 1. Introduction

Sepsis is a systemic threatening severe inflammatory status that results in multiple organ failure. It is the leading cause of death in severely ill patients, with 5.3 million deaths, and 35 million new cases of sepsis every year [1,2]. The mortality rate among sepsis patients with liver failure or renal injury may reach 70% in hospitalized patients [3,4]. The mortality rates among septic patients with liver failure or dysfunction ranged from 54% to 68%, which is more than the rates of mortality in septic patients having respiratory system failure or dysfunction. AKI (Acute-kidney injury) is widespread in septic patients. The annual global incidence of septic-AKI might be approximately 6 million cases with a mortality rate up to 44% in severe cases [5,6]. Globally, about 25 to 75% of all AKI patients are accompanied with septic shock or sepsis [7,8,9]. Sepsis’s pathogenesis is predominantly connected to the immunological disorders, inflammatory reaction, and various organ failures, in which the kidney and liver are crucial target organs [10].

LPS is a bacterial-derived endotoxin that elicits a powerful immune-inflammatory response in animals and hence, it is widely used as a pathogenic factor in sepsis research [11]. LPS exposure results in the TLRs activation (Toll-like receptors) particularly TLR4 which interacts with the downstream adaptor protein MyD88 (myeloid differentiation-primary response-88) resulting in activation of the downstream NF-κB signal transduction and lastly provokes the release of inflammation cytokines, such as interleukins (ILs) and TNF-α (tumour-necrosis factor-alpha) [12]. Moreover, one of the key characteristics of LPS-induced macrophages is the excess expression of NO (nitric oxide) synthase, particularly iNOS (inducible-nitric oxide synthase), which ultimately results in excessive NO production and subsequently inflammation [13].

Oxidative stress is another important player in the pathogenesis of sepsis that closely interplays with the inflammatory response to exacerbate cellular damage [14]. Previous studies have demonstrated the activation of apoptotic pathways during LPS-induced injuries [15,16]. New bioactive compounds that can modulate this pathogenic triangle can be more effective in controlling endotoxin-induced organ injury.

*Allium cepa* L. (common onion, Egyptian onion; family Alliaceae) is an old commercial vegetable and essential spice in various cuisines [17,18,19]. Traditionally, the plant is utilized as a remedy for diverse illnesses such as digestive disorders, insect bites, tumours, worms, and metabolic, heart, and skin diseases [20,21] and it demonstrated varied bioactivities [21,22]. This plant is an abundant source of flavonoids, including quercetin and its derivatives that were proven to demonstrate various pharmacological benefits, including antioxidative, cardio-protection, antidiabetic, anti-HIV, anti-inflammation, and anti-cancer capabilities [22,23,24,25,26,27,28].

Waste management can participate, whether indirectly or directly, in the achievement of specific targets of different SDGs (Sustainable_development_goals), whereas “good health and well-being” and “sustainable cities and communities” are the ones that are greatly affected by waste management [29]. The reuse of food industry wastes leads to the manufacture of various products with added values [30]. In this regard, onion outer skins as a waste product from the food industry represent a natural source of worthwhile functional components, which need potential ways for their utilization. In this work, two flavonoid dimers; cepabiflas B and C (CBs) were isolated from *A. cepa* outer skins that were only reported by Vu et al. [31]. This study proposed to systematically investigate their protective effects and potential molecular mechanism in septic acute hepatorenal injury. Moreover, the impact of CBs on the crosstalk of inflammatory and apoptotic pathways was investigated.

## 2. Materials and Methods

### 2.1. General

NMR analyses were performed on Bruker BioSpin GmbH 800 MHz Ultrashield spectrometer with standard Bruker software. Linear ion trap mass spectrometer Thermo Scientific LTQ-XL coupled with Accela pump and Accela autosampler (San Jose, CA, USA) was used for electrospray ionization mass spectrum measurement. A pre-coated silica gel 60 F254 TLC plates (0.2 mm, Merck, Rahway, NJ, USA) was utilized for TLC. Chromatographic investigation was performed utilizing Sephadex LH-20/silica gel 60/RP-18 (Merck). The TLC solvent systems were CHCl_3_/MeOH (I: 85/15 and II: 75/25).

### 2.2. Plant Materials

The outer scaley leaves of yellow onion were collected in June 2021 from Al-Qassim governorate, KSA. The staff members of the Department of Natural Products and Alternative Medicine, Faculty of Pharmacy, King Abdulaziz University confirmed the plant’s authenticity. A voucher specimen was kept in the herbarium of the Department under the registration number AC-2021-A.

### 2.3. Extraction and Isolation

Air-dried outer scaley leaves (5.0 kg) were extracted using MeOH (4 × 25 L) at room temperature and the combined extract was concentrated under reduced pressure using Büchi Rotavap RE111 Rotary Evaporator (50 °C water bath temperature; rotation 100–150 rpm; vacuum 250 mmHg) to afford a dark brown residue (670 g). The MeOH extract was dissolved in water and successively partitioned with n-hexane, CHCl_3_, and EtOAc. The EtOAc-soluble fraction (69 g) was subjected to silica gel column (500 g × 100 cm × 5 cm, CHCl_3_/MeOH) to obtain 10 subfractions (ACE1-ACE10) by increasing 10% MeOH for each fraction. Subfractions ACE-5 and ACE-6 (4.7 g) were collected based on TLC plates and subjected to Sephadex LH-20 CC (100 g × 50 cm × 5 cm) using MeOH as an eluent to afford six fractions. Fractions 3 to 5 were gathered and chromatographed on RP-18 (150 g × 50 cm × 3 cm) CC using H_2_O/CH_3_OH (80:20 to 40:60) that gave impure CBs, which were further purified by Sephadex LH-20 CC with MeOH elution to afford CBs (286 mg).

### 2.4. Animal Experiments

Male BALB/c mice (20–25 g, 5-week-old) were held under standard conditions prior to and throughout the experimental period. The study protocol was approved by the Batterjee Medical College Research Ethical Committee (no. RES-2022-0064), which adheres to the NIH regulations for the care and use of experimental animals.

Animals were randomly divided into five groups (6 mice/group): control mice which were given sterile saline; positive control group (CBs group) that was administered CBs (dissolved in H_2_O) (60 mg/kg, orally) for 5 days; LPS (Sigma-Aldrich, St. Louis, MO, USA) group (10 mg/kg, i.p.); two CBs + LPS treated groups that received CBs group (40 and 60 mg/kg, orally) for 5 days prior to LPS challenge (10 mg/kg/i.p.). LPS dose was selected based on previous studies [16,32] while the doses of CBs were selected based on a preliminary experiment. Five doses of CBs (5, 10, 20, 40, and 60 mg/kg) were tested against LPS induced hepatic and kidney damage. Serum markers of injury along with the histopathology of both organs were used to select the most effective doses to complete the study.

Twenty-four hours after the LPS injection, mice were euthanized by cervical dislocation under anaesthesia. Samples of blood, liver, and kidney were harvested. Serum was separated after centrifugation of blood samples and kept at −80 °C till further analysis. A part of each organ tissue was homogenized in PBS (phosphate-buffered saline) and centrifuged to obtain the supernatants for ELISA. Other pieces of each organ were submitted for histological, IHC (immune-histochemical), biological, and RT-PCR analysis.

For survival analysis, mice were divided into another 4 groups (each = 10 mice) as follows: control, CBs 60, LPS, and CBs 60 + LPS. Mice were treated with CBs (60 mg/kg) once a day for 5 days followed by LPS injection. LPS (10 mg/kg, intraperitoneally) was injected. The mice were kept and monitored every 6 h for lethality for 3 days. The percent of survival was calculated according to the death number.

### 2.5. Serum Indices of Hepatic and Renal Injury

Aminotransferases (ALT and AST), creatinine, and BUN (blood-urea nitrogen) were estimated in serum according to the colorimetric kits’ instructions (Human, Wiesbaden, Germany) using spectrophotometer.

### 2.6. Oxidative and Antioxidant Parameters

A piece (≈50–100 mg) of the liver and kidney tissue was homogenized in ice-cold buffer (EDTA 1 mM, potassium phosphate 50 mM, PH 7.5) and then centrifuged (3000× *g*/10 min/4 °C) to obtain the supernatants which were retained at 4 °C for oxidative stress and antioxidants assay using commercial kits as follows:

#### 2.6.1. Malondialdehyde (MDA, Abcam/Cambridge/UK, Cat No: ab233471)

It relies on the formation of a coloured product generation by reaction with thiobarbituric acid, which was colorimetrically estimated at 532 nm by UV spectrophotometer (T80 + UV/VIS Spectrometer PG Instruments Ltd./Lutterworth/UK). The kit sensitivity is >0.1 nmol with no significant cross-reactivity.

#### 2.6.2. 4-Hydroxynonenal (4-HNE, MyBiosource/USA, Cat No: MBS027502)

Based on the ELISA-kit manual (the kit sensitivity is 1.0 μmol/L with no significant cross-reactivity), the samples were mixed with HRP-conjugate reagent and covered with a closure plate membrane at 37 °C for 60 min then washing for four times. Finally, the chromogen solution was added and protected from light at 37 °C for 15 min, then a stop-solution was added, and optical density was assessed at 450 nm.

#### 2.6.3. Total Antioxidant Capacity (TAC, Sigma-Aldrich/St. Louis/MO/USA, Cat No: MAK187.1KT)

The samples were mixed with Cu^2+^-working solution and incubated at 25 °C/for 90 min, then the absorbance was estimated at 570 nm.

#### 2.6.4. Superoxide Dismutase (SOD, Abcam/Cambridge/UK, Cat No: ab65354)

The samples were mixed with both enzyme working solution and WST-1 and incubated for 20 min/37 °C to form a formazan dye which was assessed by absorbance increase at 450 nm. The more SOD activity in the sample, the lower the formazan dye is produced. The kit sensitivity is 0.46 ng/mL.

#### 2.6.5. Reduced Glutathione (GSH, Calbiochem/MERCK Millipore/Darmstadt/Germany, Cat No: 354102,100T)

GSH estimation depends on a reaction among 4-chloro-1-methyl-7-trifluromethyl-quinoliniumm-ethylsulfate and all mercaptans (RSH) that are existed in the supernatants, followed by β-elimination reaction under alkaline condition (NaOH 30%), producing a chromophoric thione with absorbance maxima at 400 nm. The kit sensitivity is 5.0 µM.

### 2.7. Histology

Hepatic and renal tissues were fixed with neutral formalin, dehydrated in ethanol, and then embedded in paraffin. The blocks were sliced and stained with haematoxylin and eosin. Specimens were blindly examined in random order. Lesions were graded as previously described [33,34,35]. The hepatic lesions were scored based on the presence of cytoplasmic vacuolation, pyknosis, inflammatory infiltration, congestion, and necrosis as follows: Score 0 = normal, 1 = very mild injury with cytoplasmic vacuolation, pyknosis, occasional necrosis and inflammation, 2 = ≤30% mild injury with marked pyknosis, lobular necrosis/inflammation, 3 = ≤60% moderate injury with lobular necrosis/inflammation, and 4 = severe injury with ˃60% lobular necrosis/inflammation. For kidney sections, the score was based on the presence of loss of brush border, tubular necrosis, cast formation, and tubular dilatation as follows: Score normal = 0, small focal damage = 0.5, <10% cortical damage = 1, 10–25% cortical damage = 2, 25–75% cortical damage = 3 and >75% cortical damage = 4.

### 2.8. Immunohistochemistry

The liver paraffin sections were dewaxed and processed as previously described [34,36]. Sections were IHC stained using the primary antibodies: rabbit-polyclonal-antibody against NF-kB p65 (1:200), Nrf2 (1:200) (Fisher-Scientific Inc., Waltham, MA, USA), Bcl2 (1:200), and caspase-3 (1:200) (Elabscience Biotechnology Inc., Houston, TX, USA). Diaminobenzidine (DAB) was used for visualization.

### 2.9. ELISA

NF-κB, cytokine concentrations (IL-6 and -1β and TNF-α), HO-1 (heme-oxygenase), and apoptosis parameters (Bcl-2, Bax, and caspase-3) were measured in the supernatants employing ELISA kits following the manufacturer’s instructions (Cusabio_Biotech Co., Shanghai, China, cat no. CSB-E12108m (Sensitivity 0.078 ng/mL); CSB-E04741m (Sensitivity 3.9 pg/mL); CSB-E04639m (Sensitivity 3.9 pg/mL); CSB-E08054m (Sensitivity 7.8 pg/mL); CSB-E08268m (Sensitivity 7.8 pg/mL); CSB-E08855m (Sensitivity 3.9 pg/mL); CSB-E17114m (Sensitivity 1.95 pg/mL); CSB-E08858m (Sensitivity 0.078 ng/mL), respectively). Nrf2-binding capacity was assessed in the nuclear extract as described in the kit’s guidelines (Active_Motif Inc., Carlsbad, CA, USA, cat no. 50296).

### 2.10. NO (Nitric Oxide) Estimation

This was measured according to the kit`s protocol (Bio-Diagnostic Co., Giza, Egypt, cat no. 25 33; the kit sensitivity is 2.5 μM). In brief, the tissue was homogenized utilizing an ice-cold buffer having EDTA (2 mM) before centrifugation (4000× *g*/10 min/4 °C). The supernatants and sulphanilamide and N-(1–naphthyl) ethylenediamine were mixed to produce a reddish-purple product that was spectrophotometrically quantified at 540 nm.

### 2.11. RT-PCR

The expression of TNF-α, IL-6, IL-1β, iNOS, Bcl2, caspase-3, Bax, Nrf2, and HO-1 were determined using RT-PCR. In brief, RNA was extracted using QIAzol reagent (Qiagen/Germany) according to manufacturer guidelines and then its concentration was estimated using the NanoDrop-2000 (ThermoScientific, Waltham, MA, USA). RNA (≈1 µg) was reverse transcribed utilizing the Bioline cDNA-synthesis kit (cat no. BIO-65054, Bioline, Taunton, MA, USA). RT-PCR equipment (Pikoreal 96/ThermoScientific, Waltham, MA, USA) was utilized to replicate cDNA templates. The amplification process consisted of a total volume mixture (20 µL) [10 µL of HERA SYBR green PCR Master Mix (cat no. WF10304002No/Lo/Hi, Willowfort, West Midlands, UK), 2 µL of cDNA template, 2 µL (10 pmol/µL) of each gene primer, and 6 µL of nuclease-free water], and was performed using the following program: 95 °C for 2 min, followed by 40 cycles of 95 °C for 10 s, and 60 °C for 30 s. The studied genes` primers were designed using Primer3Plus software [http://www.bioinformatics.nl/cgi-bin/primer3plus/primer3plus.cgi] (accessed on 16 January 2023). Their specificity was determined using Primer-BLAST program (NCBI/primer-BLAST [https://www.ncbi.nlm.nih.gov/tools/primer-blast/] (accessed on 3 February 2023). Primer sets were synthesized by Vivantis (Vivantis Technologies/Shah Alam/Malaysia). GAPDH (glyceraldehyde-3-phosphate dehydrogenase) was used as a control gene, and the sequences of the used primer pairs are listed in Table 1. Relative gene expression levels were represented as ∆Ct = Ct target gene − Ct control gene; fold change of gene expression was calculated according to the 2^−∆∆CT^ method.

### 2.12. Data Analysis

Shapiro–Wilk test was utilized to analyse the data normality before selecting the parametric or non-parametric tests. One-way ANOVA (analysis of variance) followed by Tukey Kramer’s multiple comparisons test were utilized for comparing the presented data (mean ± SEM). Survival curve (Kaplan–Meier curve) was analysed using the log-rank test (GraphPad Software Inc., San Diego, CA, USA). *p*-value < 0.05 indicated a significant difference.

## 3. Results

### 3.1. Purification and Characterization of Cepabiflas B and C (CBs)

**Figure 1:**
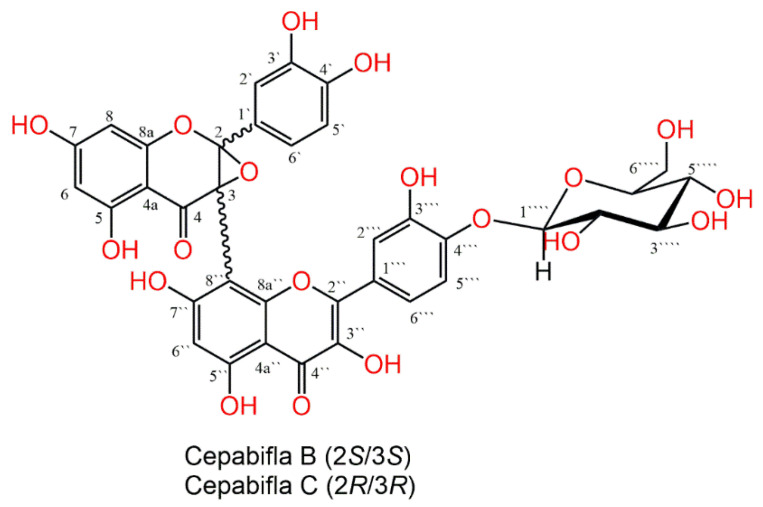
Chemical structures of cepabiflas B and C (CBs).

The MeOH extract of the outer skins of yellow onion (*A*. *cepa*) was partitioned among *n*-hexane, CH_3_Cl, and EtOAc. The combined CH_3_Cl and EtOAc fractions were separated on SiO_2_ and Sephadex LH-20 CC to afford cepabiflas B and C (CBs) as a mixture (1:1) (Figure 1). Their structures were assigned utilizing NMR and ESIMS spectral tools and comparison with the literature. The LCMS investigation demonstrated that the percentage of CBs was 0.427% in the extract (See Supplementary Materials). It is noteworthy that these compounds were separated by Vu et al. as a new metabolite in 2020 from the outer skins of red onion (*A. cepa*) obtained from Daejeon, Korea [31], and here is the second report for their isolation and characterization.

### 3.2. CBs Increased the Survival Rate and Ameliorated Serum and Histopathological Indices of Hepatorenal Damage in LPS-Intoxicated Mice

There was no notable difference between the CBs group and control in all the estimated parameters.

**Figure 2:**
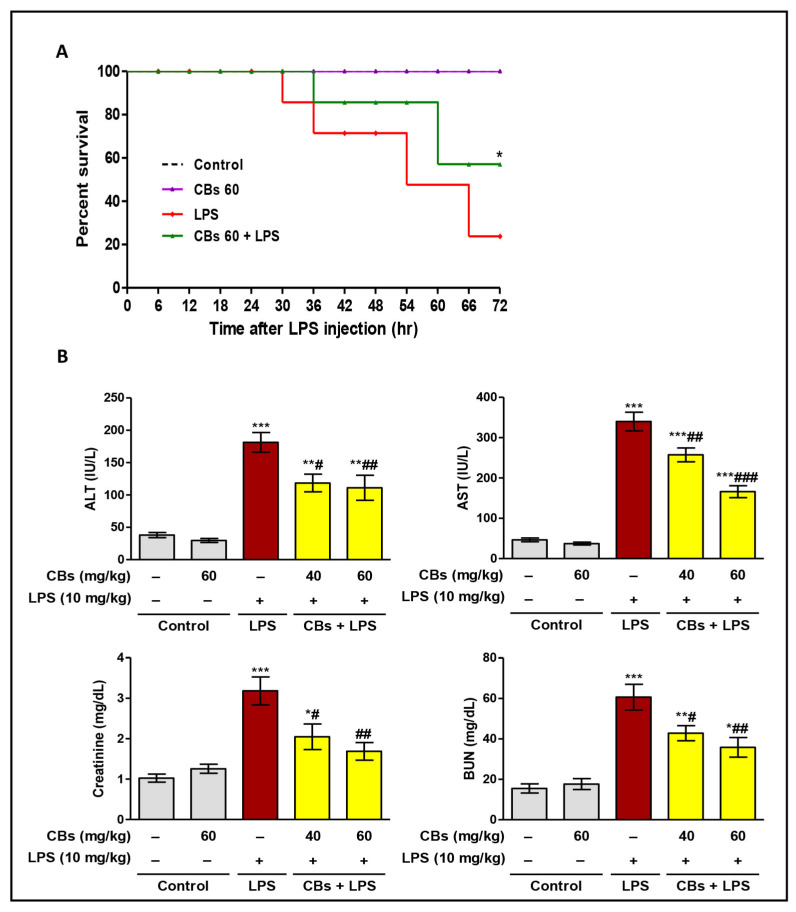
CBs increased survival rate and attenuated serum markers of hepatorenal toxicity in LPS−intoxicated mice. (**A**) Survival rate (for 72 h) after injecting LPS (10 mg/kg). (**B**) Alanine aminotransferase (ALT); Creatinine; Aspartate aminotransferase (AST); Blood urea nitrogen (BUN). Data are the mean ± SE (n = 6). * *p* < 0.05; ** *p* < 0.01; *** *p* < 0.001 vs. control group; # *p* < 0.05, ## *p* < 0.01, ### *p* < 0.001 vs. LPS group (one-way ANOVA).

LPS significantly decreased the survival rate comparing to the control group. CBs pre-treatment resulted in notable increase in survival rate compared to the LPS group (Figure 2A). LPS challenge led to a significant increase (*p* < 0.001) in serum transaminases, creatinine, and BUN compared to normal mice (Figure 2B). These indices were supported by the histopathological results.

**Figure 3:**
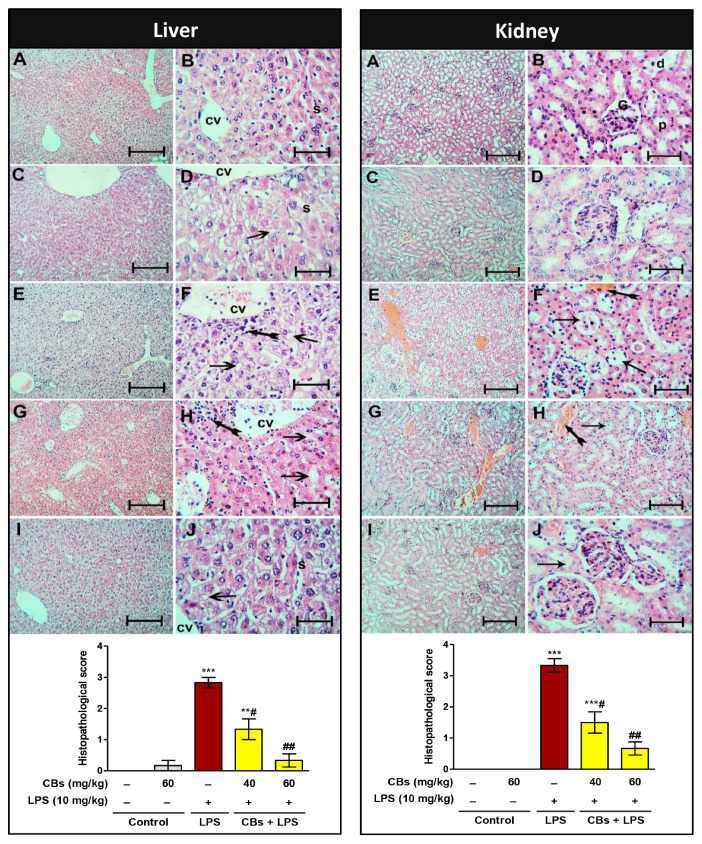
CBs alleviated LPS-induced hepatorenal histopathological damage. Examination of the liver and kidney’s histopathology using H&E staining; Panel of liver sections showed normal liver architecture of the control group with the plated hepatocytes separated by the sinusoidal spaces (S) radiating from the central veins (cv). In LPS group, hydropic de-generative alterations in the hepatocytes with complete sinusoids’ disappearance. Near the central veins, there is a noticeable lymphocytic infiltration of the liver tissue (tailed arrows). CBs pre-treated groups showed marked improvement in the hepatic lesions as the hydropic degeneration of the hepatocytes and lymphocytic infiltration were minimal. The sinusoids reappeared in the CBs 60 + LPS group. Panel of the kidney sections showed normal renal cortex of the control (**A**,**B**) and CBs (**C**,**D**) groups containing the proximal (p), glomeruli (G), and distal (d) convoluted tubules with clear lumens and homogenous acidophilic cytoplasm of the lining endothelium. LPS group (**E**,**F**) shows vacculation of the cytoplasm of the endothelial cells (arrows) and obvious vascular congestion (tailed arrow). CBs 40 + LPS group (**G**,**H**) shows minimal vacculation of the endothelium and minimal congestion. CBs 60 + LPS group (**I**,**J**) group is close the control group apart from minimal vacculation of the cytoplasm of some proximal renal tubules ((**A**,**C**,**E**,**G**,**I**) scale bar = 400 µm, (**B**,**D**,**F**,**H**,**J**) scale bar = 25 µm); Scores of histopathological hepatic and renal injury. The hepatic lesions were scored based on the presence of cytoplasmic vacuolation, pyknosis, inflammatory infiltration, congestion, and necrosis. For kidney sections, the score was based on the presence of loss of brush border, tubular necrosis, cast formation, and tubular dilatation. Data are the mean ± SE (n = 6). ** *p* < 0.01, *** *p* < 0.001 vs. control group; # *p* < 0.05, ## *p* < 0.01 vs. LPS group (one-way ANOVA).

The histopathology of the liver and kidney showed normal architecture in the control and CBs groups. On the contrary, the LPS group exhibited pathological lesions in the form of necrosis, inflammatory, and apoptotic changes (Figure 3). Notably, CBs-pre-treated groups showed a significant reduction in the abovementioned serum induces of hepatorenal injury compared to the LPS group. In addition, the histopathology of both organs showed a remarkable improvement and significant reduction in the histopathological score.

### 3.3. CBS Repressed LPS-Induced Inflammatory Response in the Liver and Kidney

**Figure 4:**
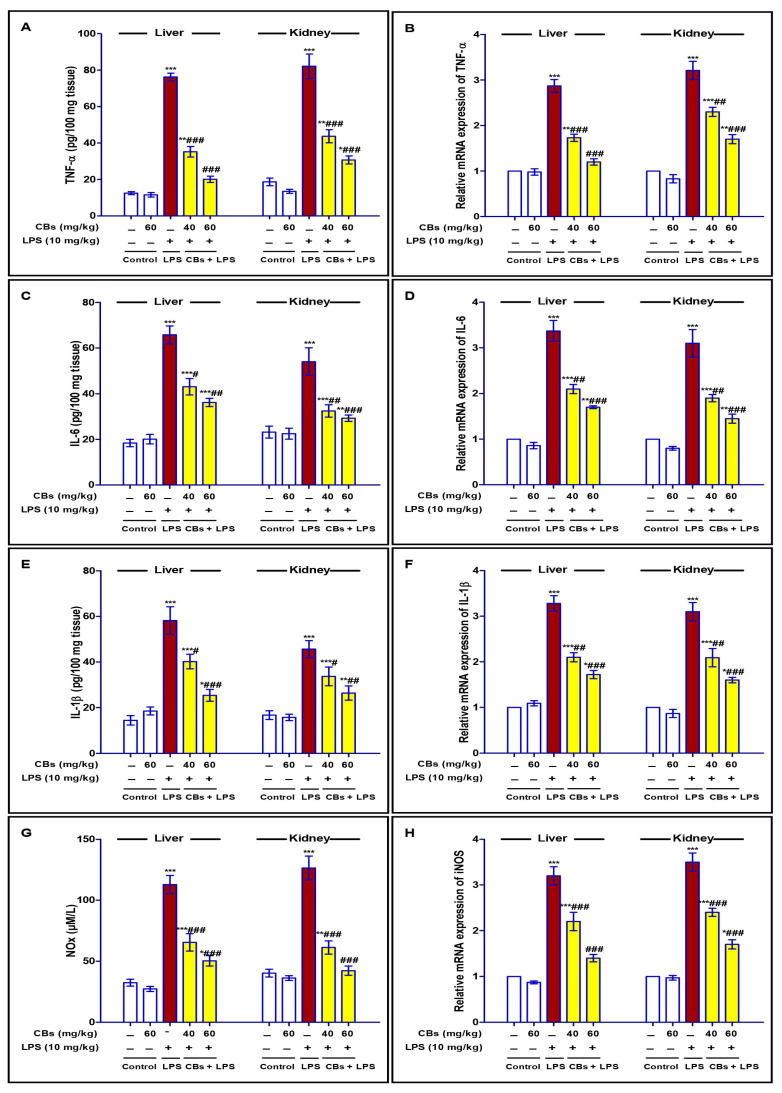
CBs repressed LPS-induced inflammatory response in the liver and the kidney. The level (**A**,**C**,**E**,**G**) and the mRNA expression (**B**,**D**,**F**,**H**) of inflammatory cytokines TNF-α, IL-6 and 1β and NO_x_. Data are the mean ± SE (n = 6). * *p <* 0.05, ** *p <* 0.01, *** *p <* 0.001 vs. control group; # *p <* 0.05, ## *p <* 0.01, ### *p <* 0.001 vs. LPS group (one-way ANOVA).

LPS injection resulted in a significant elevation (*p* < 0.001) in the expression and consequently the levels of inflammatory cytokines (TNF-α, IL-6 and 1β, and NO_x_) in the hepatic and kidney tissues compared to that of the normal mice. However, CBs treatments prior to the exposure to the LPS challenge efficiently repressed these significant rises in cytokines, especially at the dose level of 60 mg/kg (Figure 4).

### 3.4. CBS Alleviated LPS-Induced Activation of NF-κB in the Liver and Kidney

**Figure 5:**
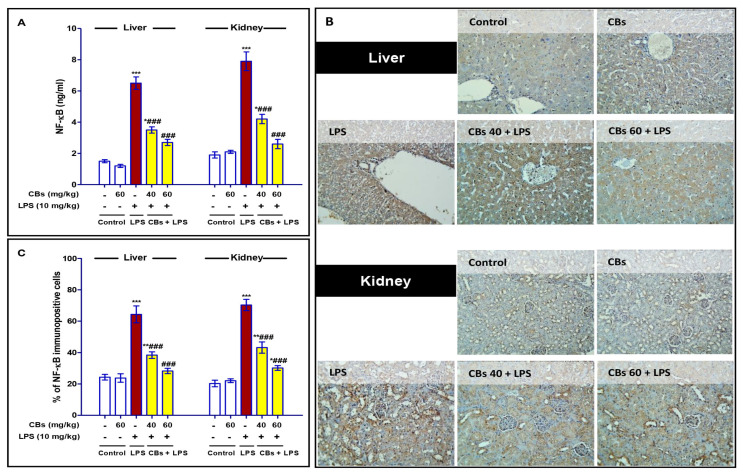
CBs alleviated LPS-induced activation of NF-κB in liver and kidney. (**A**) Level of NF-κB in the hepatic and kidney tissue; (**B**) NF-κB immunostaining of the hepatic and kidney tissue where NF-κB was intensified in the LPS group while CBs pretreated groups exhibited much lower immuno-stain; (**C**) The % of NF-κB immuno-positive cells in the hepatic and kidney tissue. Data are the mean ± SE (n = 6). * *p <* 0.05, ** *p <* 0.01, *** *p <* 0.001 vs. control group, ### *p <* 0.001 vs. LPS group (one-way ANOVA).

The inflammatory cascade that occurs during endotoxin-induced injury is closely linked to NF-κB signalling. Our results showed significant increase in the level and immuno-expression of NF-κB in the liver and the kidney compared to control mice that was declined in case of CBs pre-treatment (Figure 5).

### 3.5. CBS Inhibited LPS-Induced Apoptosis of the Liver and Kidney

**Figure 6:**
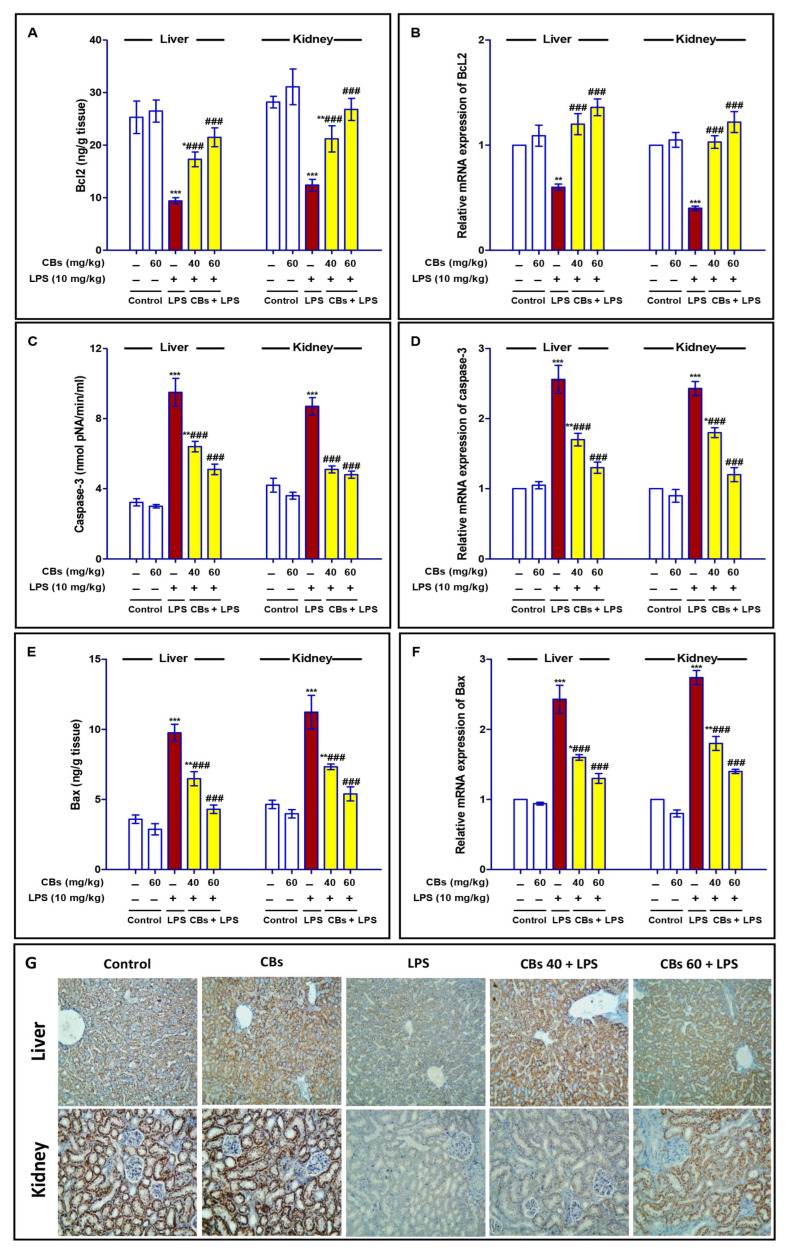

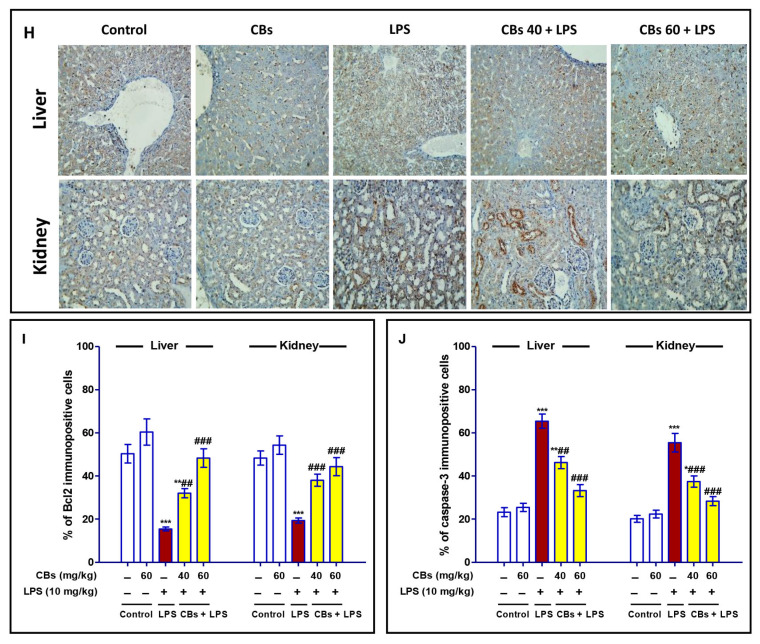
CBs counteracted LPS-induced apoptosis in liver and kidney. (**A**–**F**) Level and mRNA of Bcl2, caspase-3 and Bax in liver and kidney among different animal groups. (**G**) The protein immuno-expression of Bcl2 in liver and kidney, the stain was minimal in LPS group while CBs pretreatment enhanced Bcl2 expression. (**H**) The protein immuno-expression of caspase-3 in liver and kidney among different animal groups. (**I**,**J**) % of immuno-positive cells of Bcl2 and caspase-3. Data are the mean ± SE (n = 6). * *p <* 0.05, ** *p <* 0.01, *** *p <* 0.001 vs. control group, ## *p <* 0.01, ### *p <* 0.001 vs. LPS group (one-way ANOVA).

As shown in Figure 6, the LPS challenge resulted in a marked decrease (*p* < 0.001) in the mRNA expression, level, and number of hepatocytes positive for Bcl2 in the liver and kidney.

Furthermore, there was a marked increase (*p* < 0.001) in mRNA expression, level, and the immuno-stain for cleaved caspase 3 in LPS group. The mRNA expression of Bax as well as its level were significantly augmented in the LPS group. On the other hand, CBs pre-treatments abated all these changes. CBs enhanced the mRNA and protein expression of Bcl2, and consequently increased its level compared to LPS group. On the contrary, CBs suppressed the apoptotic parameters. CBs pre-treatment inhibited the increase in the expression and level of caspase-3 and Bax comparing to the LPS group.

### 3.6. CBS Reversed LPS-Induced Oxidative Stress and Enhanced the Antioxidants in the Liver and the Kidney

In comparison to normal mice, LPS injection led to a significant increase (*p* < 0.001) of lipid peroxidation markers (4-HNE and MDA) concurrent with a significant reduction in the content of the antioxidant enzymes (TAC, SOD, and GSH) in the liver and kidney tissues (Table 2).

Noteworthy, CBs pre-treatment reversed the increase in 4-HNE and MDA contents and enhanced the antioxidant enzymes compared to the LPS group.

### 3.7. CBS Enhanced Nrf2 Signalling in the Liver and the Kidney of LPS-Intoxicated Mice

**Figure 7:**
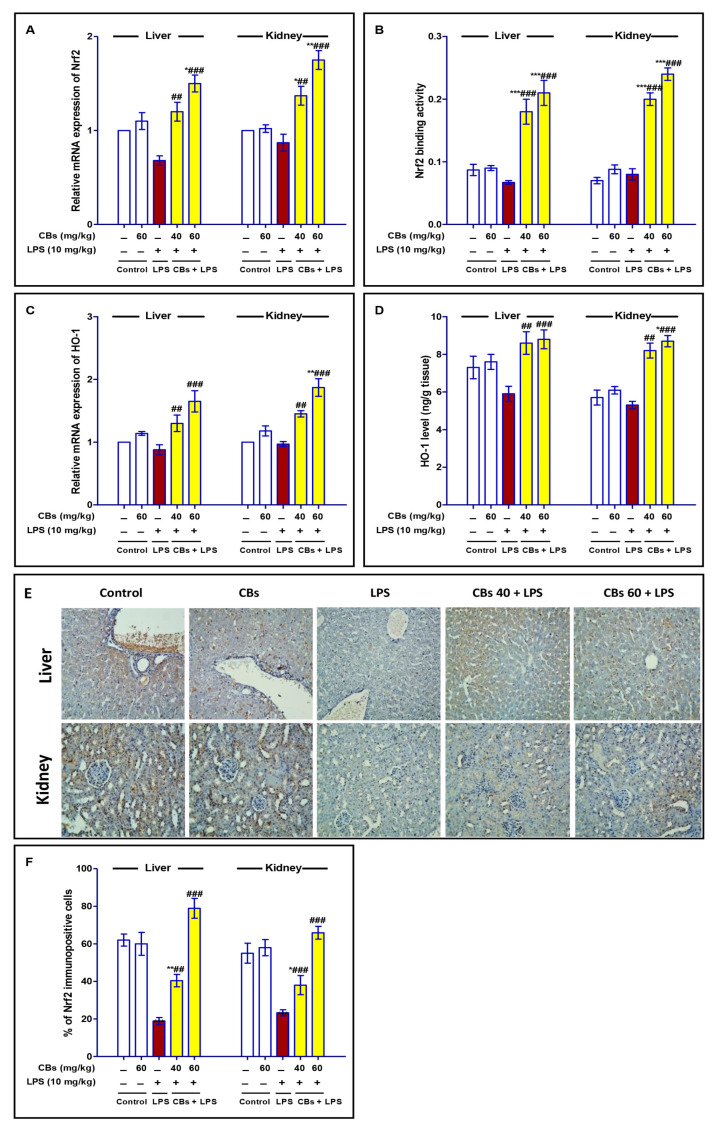
CBs enhanced Nrf2 signalling in the liver and the kidney of LPS-intoxicated mice. (**A**,**B**) mRNA expression of Nrf2 and its binding activity. (**C**,**D**) mRNA expression and level of HO-1 in hepatic and kidney tissues. (**E**) Immuno-expression of Nrf2 in the liver and kidney tissue where specimen of LPS group showed decreased immuno-stain compared to the control group while specimen of CBs pre-treated groups exhibited enhanced Nrf2 immuno-stain. (**F**) % of immuno-positive cells of Nrf2. Data are the mean ± SE (n = 6). * *p* < 0.05, ** *p* < 0.01, *** *p* < 0.001 vs. control group; *p* < 0.05, ## *p* < 0.01, ### *p* < 0.001 vs. LPS group (one-way ANOVA).

As presented in Figure 7, LPS induced a non-significant decrease in the mRNA expression and binding activity of Nrf2, in addition to mRNA expression and level of HO-1. However, CBs pre-treatment significantly enhanced Nrf2 mRNA expression, its binding activity, mRNA expression of HO-1 and its level compared to the LPS group. Additionally, the immunostaining of Nrf2 was greatly enhanced in CBs pre-treated groups.

## 4. Discussion

Sepsis results in deleterious damage to many of the body organs such as the kidneys and the liver. LPS-induced cellular damage leads to the failure of these vital organs. Treatment of sepsis and its associated injurious effects attract the attention of many researchers to establish a new therapeutic strategy with minimal side effects. Based on the previous reports on the anti-inflammatory activity of onion and its flavonoid [37], the current study was designed to explore the protective effect of CBs (new dimeric flavonoid glucosides) on hepatorenal damage in endotoxic mice. The results have demonstrated the potent ability of CBs to counteract the deleterious effects of LPS on both organs. These effects were linked to CBs’ antioxidant, anti-inflammatory, and anti-apoptotic activities via the activation of Nrf2, inhibition of NF-κB signal transduction, and modulation of apoptotic pathways.

Various investigations reported the protective potential of flavonoids such as fisetin against LPS-induced septic damage in the kidney [16], morin against LPS-induced lung injury [38], and quercetin against sepsis-linked organ impairments [39]. Moreover, onion MeOH extract was found to attenuate inflammatory mediators (e.g., NO, TNF-α, interleukins (IL)-1β, and -6) induced by LPS in BV2 microglia [40] while onion peels flavonoids and onion-prepared nanoparticles suppressed the LPS-produced NO production in BV-2 cells and RAW264 cells, respectively [41].

LPS injection results in a well-established rodent model of endotoxemia with multiple organ injury. Oxidative, inflammatory, and apoptotic reactions interconnect to induce organ injury [42]. Serum parameters such as aminotransferases indicate hepatocyte damage while creatinine and urea nitrogen are indices of renal function [43]. Following LPS injection, these parameters were elevated indicating the occurrence of hepatic and kidney damage in the setting of LPS-induced damage. These data are in line with previous studies that documented the injurious effects of LPS on the liver and kidney [14,44]. Importantly, CBs attenuated the increase in serum aminotransferases, creatinine, and BUN in LPS-challenged mice. Combined with the results of HE staining which revealed the improvement of the histopathological lesions in both organs in CBs pre-treated groups, it was concluded that CBs could significantly protect mice from LPS-induced hepatic and kidney damage.

Following LPS injection, multiple molecular pathways interplay and crosstalk to mediate LPS-induced injurious effects. LPS stimulates endothelial cells and mononuclear macrophages through the body’s cell signal transduction system, and the synthesis and release of various inflammatory mediators [45], which in turn cause a set of reactions in the body. Pro-inflammatory cytokines such as TNF-α, IL-6 and 1β, and NO_x_ play a pivotal role during LPS-induced inflammatory response [46,47]. Many studies have documented the excessive accumulation of inflammatory mediators during sepsis which exacerbates the inflammation and subsequent multiple organ failure [48]. In our research, serum pro-inflammatory factors TNF-α, IL-6 and -1β, and NO_x_ were remarkably elevated in mice injected with LPS. However, CBs decreased the level and mRNA expression of these inflammatory mediators in hepatic and kidney tissues and these findings suggested that CBs had an anti-inflammatory activity which may be responsible for its protective effects on endotoxic mice.

In terms of possible inflammatory signalling mechanisms, new studies have emphasized on the importance of NF-κB activation during sepsis and its modulating effects on the release of inflammatory cytokines [49,50,51,52]. Our data were in the same line and confirmed the NF-κB signalling activation. Notably, CBs suppressed the activation of NF-κB which was consistent with depressed inflammatory mediators. Furthermore, NF-κB activation and cytokine release are strongly linked to the apoptotic changes which play a substantial role in cell death. Previous studies had documented the upward increase in the apoptotic markers and suppression of anti-apoptotic factors following LPS injection [42,48,53]. A fact that was further confirmed by our results which showed the enhancement of Bax and caspase-3 (pro-apoptotic factors) and depression of Bcl2 (anti-apoptotic factor) in LPS group. These results suggested that CBs promoted cell survival through the suppression of apoptotic modulators as NF-κB signalling.

One of the major players that mediate LPS-induced organ injury is the generation of free radicals and associated oxidative stress. Pro-inflammatory mediators are known to exacerbate ROS generation, which can activate several intracellular signalling pathways including the one that involves the transcription factor, NF-κB [42]. Furthermore, following LPS, overproduction of ROS produces lipid peroxidative damage which leads to the destruction of the mitochondrial outer membrane, resulting in high expression of TNF-α, Bax, and caspase that induces apoptosis [54,55]. Our data confirmed this point. In LPS-mediated endotoxemia, the lipid peroxidative markers (MDA and 4-HNE) were abnormally elevated while the antioxidants (TAC, GSH, and SOD) were declined, indicating that oxidative stress damage occurred. Interestingly, CBs showed potent antioxidant efficacy and succeed to ameliorate LPS-induced oxidative stress and enhancing endogenous antioxidants in the liver and kidney tissues. This effect of CBs might be in part responsible for its beneficial protective activity against LPS-induced liver and kidney damage.

The transcription factor Nrf2 can control the expression of antioxidant response elements and cytoprotective genes and hence, it can oppose oxidative inflammatory damage. Normally, Nrf2 is repressed by Keap1 but under stress conditions, it is activated and translocated into the nucleus to stimulate the expression of various genes, including HO-1 [12]. The crucial role of the HO-1/Nrf2 signalling pathway in the mediation of LPS-induced injury in the liver and kidney was discussed previously in many investigations [12,56,57,58,59]. In line with these data, our results showed that the increased oxidative stress and restrained antioxidant capacity of the liver and the kidney were contaminant with a decrease in the protein expression of Nrf2. Interestingly, CBs facilitated the activation of Nrf2 to enhance the anti-oxidative capacity. So, it may be acceptable to suggest that CBs protective activity could be linked to its ability to potentiate Nrf2-dependent antioxidative machinery.

Clinically endotoxemia and sepsis treatment usually begin after infection and disease manifestation, which may be a limitation of this study. However, the beneficial effects of CBs treatment after the LPS challenge will be a target for further investigation.

## 5. Conclusions

**Figure 8:**
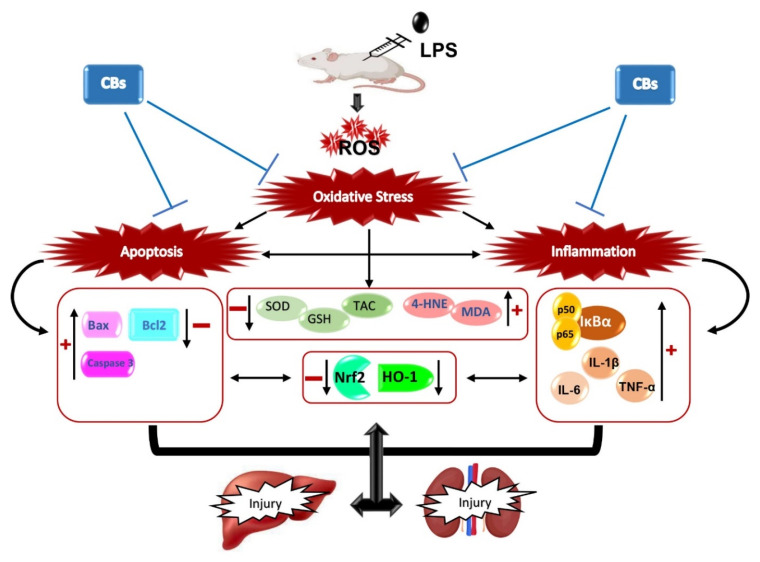
Schematic diagram of the possible mechanisms that is accountable for the protective effects of CBs against LPS-induced hepatic and kidney injury.

Overall, our results indicated that CBs isolated from yellow onion exerted potent anti-apoptotic and anti-inflammatory activities via repression of NF-κB and Bax/Bcl2 signalling which led to attenuation of LPS-associated hepatic and kidney damage (Figure 8).

All these findings suggested the possibility of developing CBs as a new anti-inflammatory molecule for septic patients. although further investigation is mandatory for full elucidation of the molecular mechanisms of CBs. Moreover, these findings further reinforced the therapeutic values of *A. cepa* and its biometabolites that could be developed into nutraceuticals and functional foods for the management and prevention of various health disorders.

## Figures and Tables

**Table 1:** The sequence of the primers employed in RT-PCR.

Gene (Mouse)	PCR Product (bp)	Sequence (5′-3′)
*TNF-α*	99	**F:** TGAACTTCGGGGTGATCGGT
		**R:** GGTGGTTTGTGAGTGTGAGGG
*IL-6*	79	**F:** AGTCCTTCCTACCCCAATTTCC
		**R:** GGTCTTGGTCCTTAGCCACT
*IL-1β*	81	**F:** GCAACTGTTCCTGAACTCAACT
		**R:** GGGTCCGTCAACTTCAAAGA
*iNOS*	75	**F:** TGGTGAAGGGACTGAGCTGT
		**R:** GCTACTCCGTGGAGTGAACA
*Bcl2*	123	**F:** CCTGTGGATGACTGAGTACCTG
		**R:** AGCCAGGAGAAATCAAACAGAGG
*Caspase-3*	74	**F:** ATGGAGAACAACAAAACCTCAGT
		**R:** TTGCTCCCATGTATGGTCTTTAC
*Bax*	140	**F:** TGAAGACAGGGGCCTTTTTG
		**R:** AATTCGCCGGAGACACTCG
*Nrf2*	170	**F:** AAGAATAAAGTCGCCGCCCA
		**R:** AGATACAAGGTGCTGAGCCG
*HO-1*	122	**F:** GAAATCATCCCTTGCACGCC
		**R:** CCTGAGAGGTCACCCAGGTA
*Glyceraldehyde-3-phosphate dehydrogenase (GAPDH)*	123	**F:** AGGTCGGTGTGAACGGATTTG
		**R:** TGTAGACCATGTAGTTGAGGTCA

**Table 2:** CBs ameliorated oxidative stress and enhanced antioxidants in hepatic and renal tissues.

Parameters	Groups				
	Control	CBs	LPS	CBs (40 mg/kg) + LPS	CBs (60 mg/kg) + LPS
**MDA (nmol/g tissue)**					
Liver	29.5 ± 3.0	22.8 ± 2.1	73.1 ± 4.9 ***	52.6 ± 4.3 **##	35.3 ± 4.0 ###
Kidney	23.6 ± 2.8	20.9 ± 2.4	65.4 ± 5.4 ***	46.6 ± 3.7 **##	28.8 ± 2.9 ###
**4-HNE (µmol/mL)**					
Liver	0.36 ± 0.05	0.31 ± 0.04	1.02 ± 0.1 ***	0.7 ± 0.06 *#	0.57 ± 0.07 ###
Kidney	0.42 ± 0.05	0.38 ± 0.05	1.24 ± 0.08 ***	0.82 ± 0.05 ***###	0.6 ± 0.04 ###
**TAC (nmol/g tissue)**					
Liver	0.75 ± 0.06	0.81 ± 0.04	0.36 ± 0.03 ***	0.57 ± 0.03 *#	0.71 ± 0.04 ###
Kidney	0.58 ± 0.08	0.67 ± 0.02	0.25 ± 0.01 ***	0.48 ± 0.03 ##	0.54 ± 0.02 ##
**SOD (U/g tissue)**					
Liver	22.6 ± 2.2	26.9 ± 1.2	7.9 ± 0.3 ***	16.4 ± 1.9 **##	18.6 ± 4.6 ###
Kidney	20.2 ± 1.9	25.1 ± 2.7	8.7 ± 0.8 ***	13.9 ± 1.5 *##	15.1 ± 2.1 ###
**GSH (µmol/g tissue)**					
Liver	14.8 ± 1.9	17.9 ± 1.5	4.7 ± 0.4 ***	8.3 ± 0.8 **##	12.3 ± 1.4 ###
Kidney	16.3 ± 1.3	14.5 ± 1.3	6.9 ± 0.2 ***	10.1 ± 1.5 **##	13.1 ± 1.1 ###

Results presented as the mean ± SEM (n = 6). * *p* ˂ 0.05, ** *p <* 0.01, *** *p <* 0.001 vs. the control, # *p* ˂ 0.05, ## *p <* 0.01, ### *p <* 0.001 vs. LPS group (ANOVA followed by Tukey-Kramer multiple comparison).

## Data Availability

Data are included within the article and the Supplementary Materials.

## Supplementary Materials

The following supporting information can be downloaded at: https://www.mdpi.com/article/10.3390/biology12070938/s1. Figures S1 and S2: ^1^H and ^13^C NMR spectra of CBs; Figure S3: LCMS chromatogram of CBs.; Figure S4. HPLC chromatogram of CBs; Table S1: NMR spectral data of CBs.
