# Supplementary material for: Cepabiflas B and C as Novel Anti-Inflammatory and Anti-Apoptotic Agents against Endotoxin-Induced Acute Kidney and Hepatic Injury in Mice: Impact on Bax/Bcl2 and Nrf2/NF-κB Signalling Pathways
Source: Biology (Basel). 2023 Jun 30;12(7):938. doi: 10.3390/biology12070938 (PMC10376508; doi:10.3390/biology12070938)
Supplement: Supplementary file 1 [file biology-12-00938-s001.zip › biology-2453806-supplementary.pdf]

Supplementary Materials

Cepabiflas B and C as Novel Anti-Inflammatory and Anti-Apoptotic Agents Against Endotoxin-Induced Acute Kidney and Hepatic Injury in Mice: Impact on Bax/Bcl2 and Nrf2/NF-κb Signalling Pathways

Akaber T. Rizq [1], Alaa Sirwi [1], Dina S. El-Agamy [2], Hossam M. Abdallah [1], Sabrin R. M. Ibrahim [3,4] and Gamal A. Mohamed [1,*]

[1] Department of Natural Products and Alternative Medicine, Faculty of Pharmacy, King Abdulaziz University, Jeddah 21589, Saudi Arabia; Aasaadrizq@stu.kau.edu.sa (A.T.R); asirwi@kau.edu.sa (A.S.); hmafifi@kau.edu.sa (H.M.A.)

[2] Department of Pharmacology and Toxicology, Faculty of Pharmacy, Mansoura University, Mansoura 35516, Egypt (dinaagamy@mans.edu.eg; dinaagamy1@yahoo.com).

[3] Department of Chemistry, Preparatory Year Program, Batterjee Medical College, Jeddah 21442, Saudi Arabia; sabrin.ibrahim@bmc.edu.sa

[4] Department of Pharmacognosy, Faculty of Pharmacy, Assiut University, Assiut 71526, Egypt

* Correspondence: gahussein@kau.edu.sa; Tel.: +966 597636182

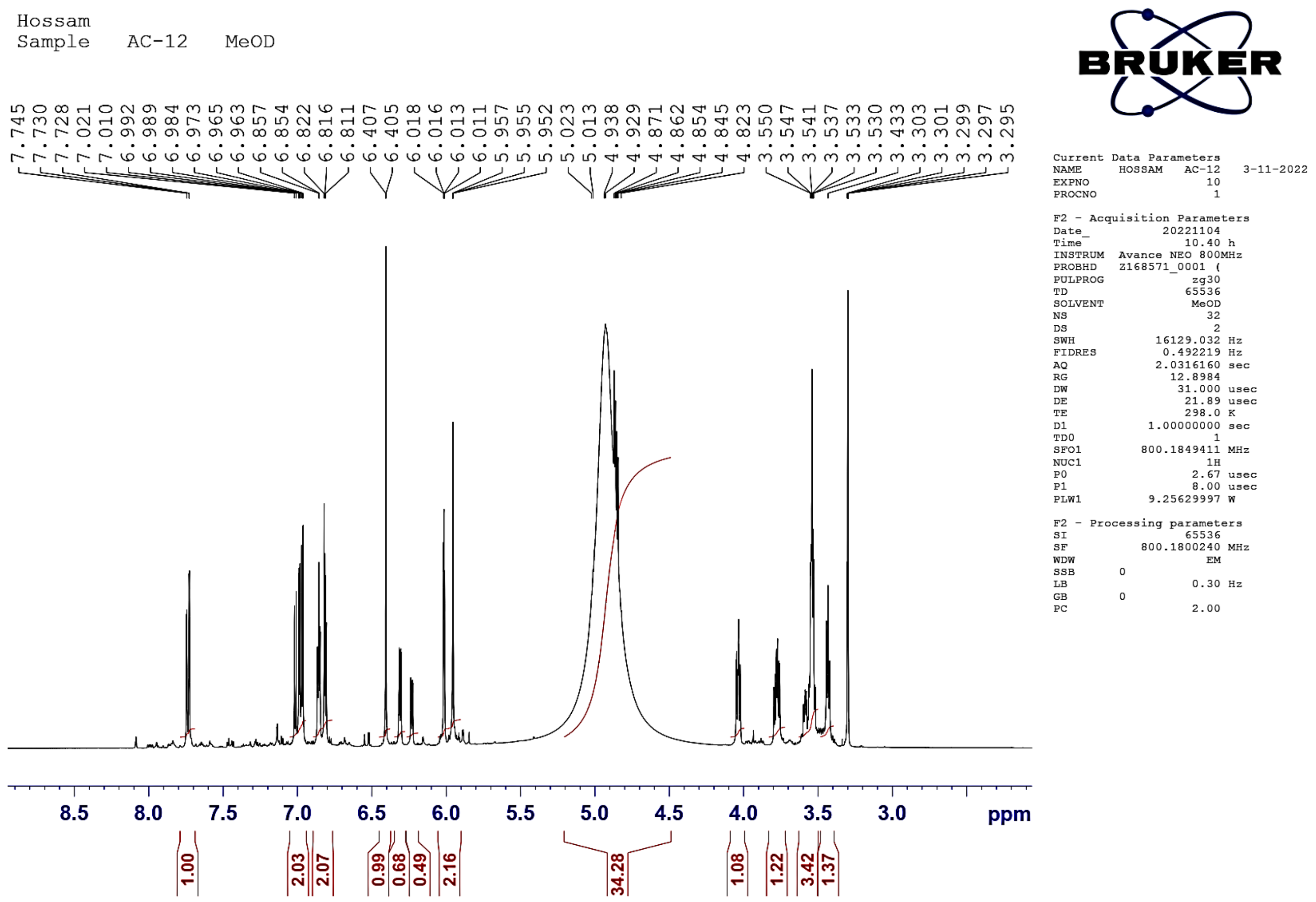

Figure S1 ^{1}H NMR spectrum (800 MHz, CD_3OD) of CBs.

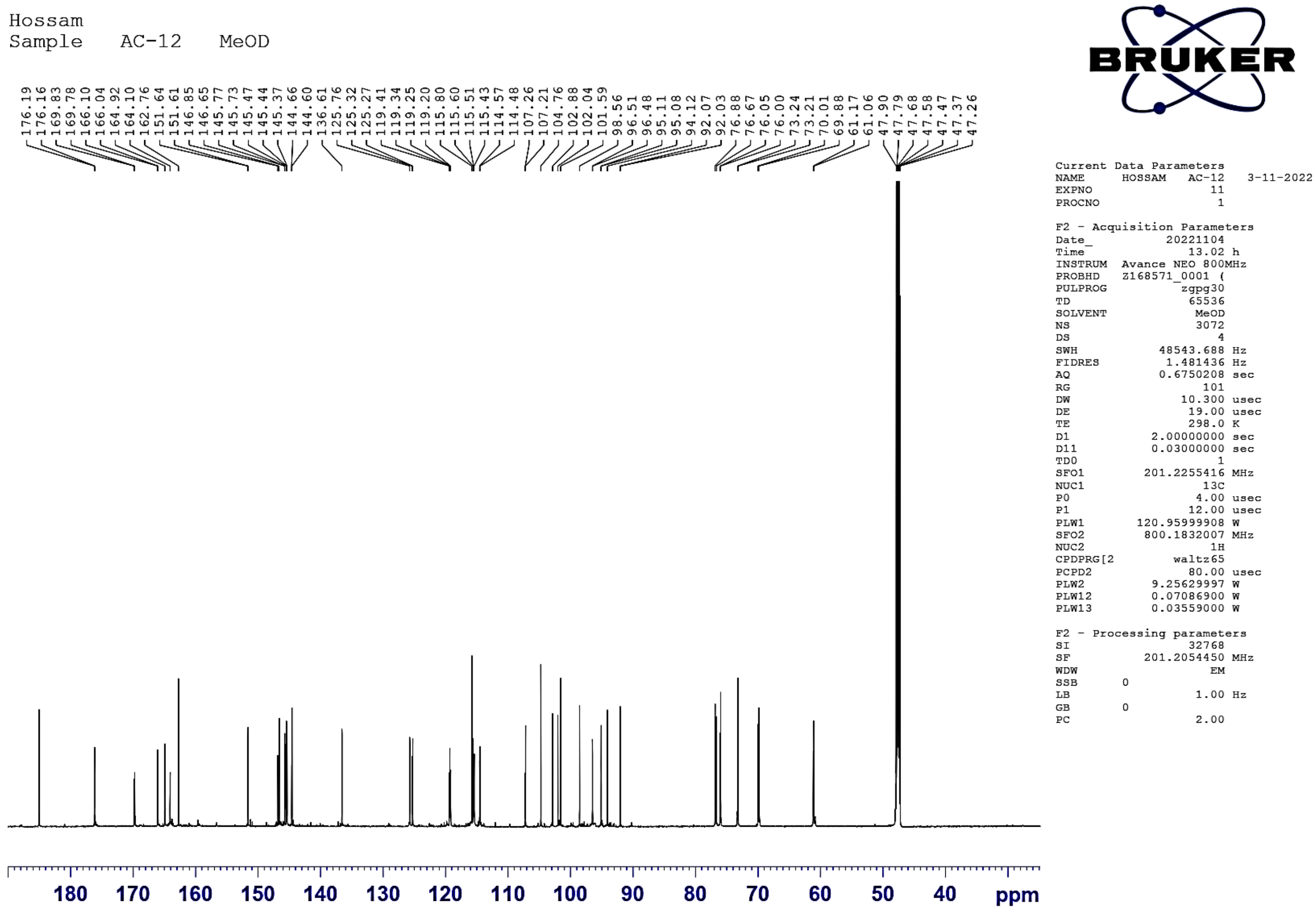

Figure S2 ^{13}C NMR spectrum (200 MHz, CD_3OD) of CBs.

Quantification of Cabiflas B and C

Mobile: line A; acetonitrile, line B; 1 L water, 1g ammonium acetate, 1 mL glacial acetic acid. Pump programmed to deliver 400 µL/min of 1: 1, v/v of A:B.

Extract prepared in methanol, 10 mg/mL, filtered through PTFE 0.22 um syringe filter and 5 uL was injected for LCMS analysis.

% of Cabiflas B and C = 0.427 % (Each 100 g extract contain 42.7 mg of CBs)

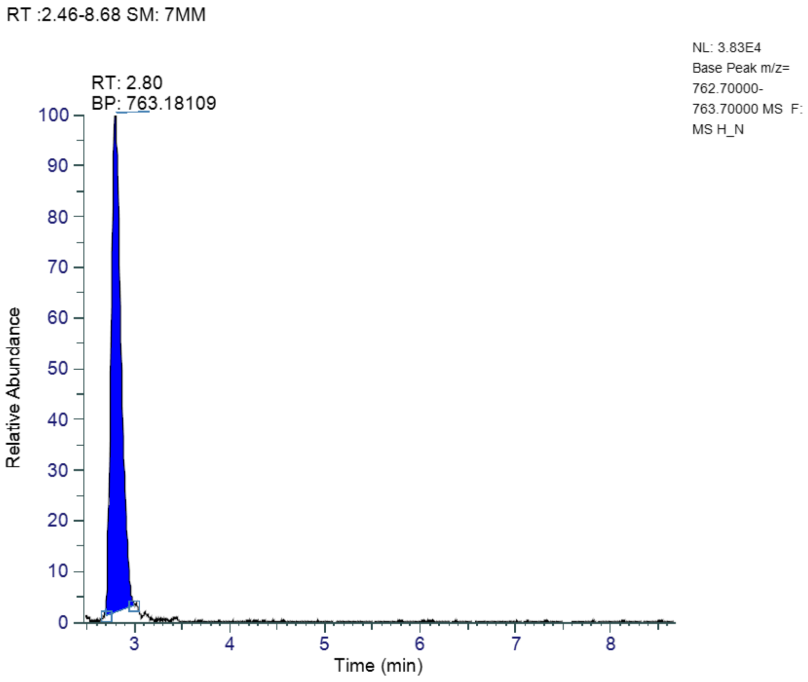

Figure 3. MRM spectrum of Cepabiflas B and C.

```
Data File C:\CHEM32\1\DATA\MIX-2023\MIX-2023000139.D
Sample Name: AC-12

=====================================================================
Acq. Operator   : SAMEH ELHADY
Acq. Instrument : Instrument 1                   Location : Vial 91
Injection Date  : 4/4/2023 11:45:19 PM
                                              Inj Volume : 1.0 µl
Acq. Method     : C:\CHEM32\1\METHODS\SAMEH-2022-23.M
Last changed    : 4/4/2023 11:45:01 PM by SAMEH ELHADY
                  (modified after loading)
Analysis Method : C:\CHEM32\1\METHODS\SAMEH-2022-23.M
Last changed    : 2/6/2023 10:51:36 AM by SAMEH
Sample Info     : 50% ACN
                  1 ML/MIN
                  Zorbax Agilant 250 X 4.6 mm
```

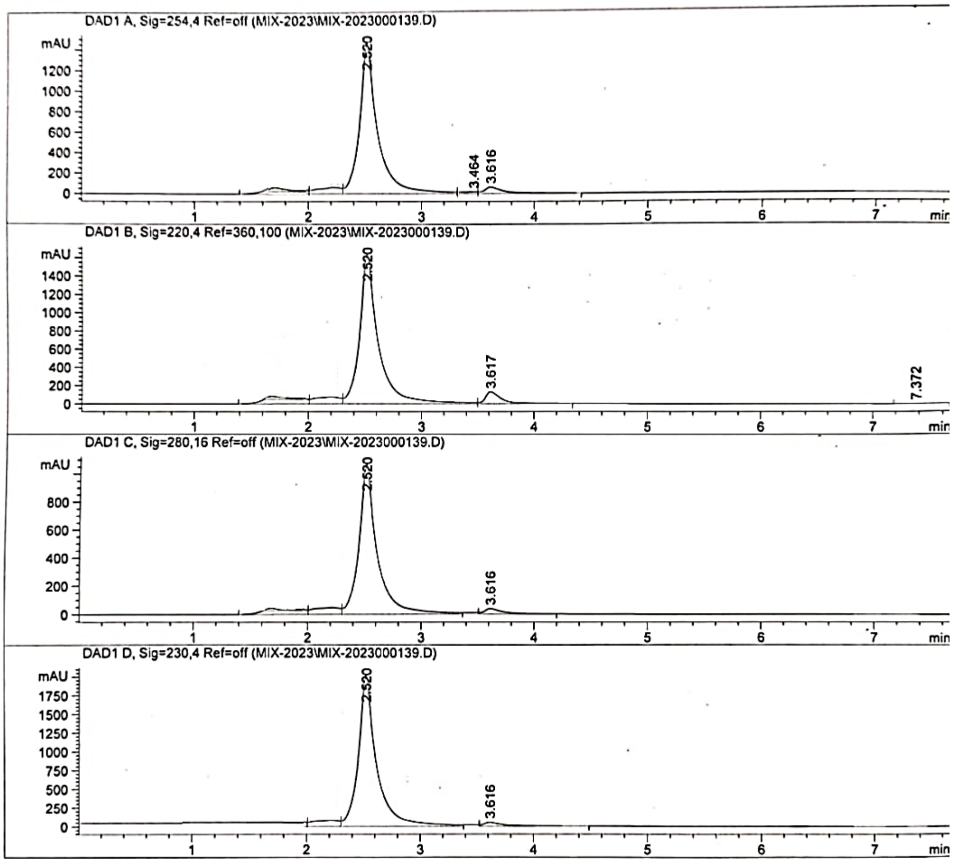

Instrument 1 4/5/2023 11:18:13 AM SAMEH-NOHA   Page 1 of 3

Figure S4 HPLC spectrum of CBs.

Table S1 NMR spectral data of cepabiflas B and C (CD_3OD, 800 and 200 MHz).

	Cepabifla B (2*S*, 3*S*)			Cepabifla C (2*R*, 3*R*)		
**No.**	**δ_H [mult., *J* (Hz)]**	**δ_C (mult.)**	**HMBC**	**δ_H [mult., *J* (Hz)]**	**δ_C (mult.)**	**HMBC**
**2**	-	92.1 C	-	-	92.0 C	-
**3**	-	102.9 C	-	-	104.7 C	-
**4**	-	185.1 C	-	-	185.1 C	-
**4a**	-	98.6 C	-		98.6 C	-
**5**	-	166.0 C	-	-	166.1 C	-
**6**	6.01 d (1.6)	95.1 CH	4, 4a, 5, 7, 8	5.95 d (1.6)	95.5 CH	4, 4a, 5, 7
**7**	-	169.8 C	-	-	169.7 C	
**8**	6.40 d (1.6)	94.1 CH	2, 4a, 6, 7, 8a	6.40 d (1.6)	94.1 CH	2, 6, 7, 8a
**8a**	-	164.9 C	-	-	164.9 C	-
**1`**	-	125.8 C	-	-	125.8 C	-
**2`**	6.99 d (1.8)	115.6 CH	2, 1`, 3`, 4`, 6`	6.97 d (1.8)	115.4 CH	2, 1`, 3`, 4`, 6`
**3`**	-	145.4 C	-	-	145.3 C	-
**4`**	-	145.5 C	-	-	145.5 C	-
**5`**	6.84 d (8.8)	114.6 CH	1`, 3`, 4`, 6`	6.80 d (8.8)	114.5 CH	1`, 3`, 4`, 6`
**6`**	6.86 dd (8.8, 1.8)	119.3 CH	2, 1`, 2`, 4`	6.82 dd (8.8, 1.8)	119.2 CH	2, 1`, 2`, 4`
**2``**	-	144.6 C	-	-	144.7 C	-
**3``**	-	136.6 C	-	-	136.6 C	-
**4``**	-	176.2 C	-	-	176.1 C	-
**4a``**	-	104.8 C	-	-	104.8 C	-
**5``**	-	162.8 C	-	-	162.8 C	-
**6``**	6.40 s	94.1 CH	4``, 4a``, 5``, 8``	6.40 s	94.2 CH	4``, 4a``, 5``, 8``
**7``**	-	164.1 C	-	-	164.0 C	-
**8``**	-	107.3 C	-	-	107.2 C	-
**8a``**	-	151.7 C	-	-	151.6 C	-
**1```**		125.3 C			125.2 C	
**2```**	7.75 d (1.6)	115.8 CH	2``, 3```, 4```, 6```	7.73 d (1.6)	115.8 CH	2``, 3```, 4```, 6```
**3```**	-	145.8 C	-	-	145.7 C	-
**4```**	-	146.9 C	-	-	146.7 C	-
**5```**	7.01 d (8.8)	115.5 CH	1```, 3```, 4```, 6```	6.98 d (8.8)	115.5 CH	1```, 3```, 4```, 6```
**6```**	6.31 dd 8.8, 2.4)	119.4 CH	2``, 1```, 4```, 5```	6.24 dd 8.8, 2.4)	119.3 CH	2``, 1```, 4```, 5```
**1````**	4.88 d (7.7)	101.6 CH	4```	4.88 d (7.7)	102.0 CH	4```
**2````**	3.54 m	73.2 CH	1````, 3````, 4````	3.54 m	73.3 CH	1````, 3````, 4````
**3````**	3.55 m	76.7 CH	1````, 5````	3.55 m	76.9 CH	1````, 5````
**4````**	3.44 m	69.9 CH	2````	3.44 m	70.0 CH	2````
**5````**	3.53 m	76.0 CH	3````, 4````	3.53 m	76.1 CH	3````, 4````
**6````**	4.03 m	61.1 CH	4````, 5````	4.03 m	61.2 CH	4````, 5````
	3.77 m			3.77 m		
